# Nerve Growth Factor Signaling from Membrane Microdomains to the Nucleus: Differential Regulation by Caveolins

**DOI:** 10.3390/ijms18040693

**Published:** 2017-03-24

**Authors:** Ambre Spencer, Lingli Yu, Vincent Guili, Florie Reynaud, Yindi Ding, Ji Ma, Jérôme Jullien, David Koubi, Emmanuel Gauthier, David Cluet, Julien Falk, Valérie Castellani, Chonggang Yuan, Brian B. Rudkin

**Affiliations:** 1 East China Normal University, Key Laboratory of Brain Functional Genomics of the Ministry of Education of PR China, Joint Laboratory of Neuropathogenesis, ECNU, ENS Lyon, CNRS, Shanghai 200062, China; ambre.spencer@gmail.com (A.S.); liliyunet@gmail.com (L.Y.); emeline.d@gmail.com (Y.D.); jma@bio.ecnu.edu.cn (J.M.); david.cluet@ens-lyon.fr (D.C.); 2 Univ. Lyon, Ecole normale supérieure de Lyon, Université Claude Bernard Lyon 1, CNRS, Differentiation & Cell Cycle Group, Laboratoire de Biologie Moléculaire de la Cellule, UMR5239, 69007 Lyon, France; vincent.guili@ac-lyon.fr (V.G.); jj256@gurdon.cam.ac.uk (J.J.); david.koubi@finovatis.com (D.K.); Emmanuel.GAUTHIER@ch-le-vinatier.fr (E.G.); 3 East China Normal University, School of Life Sciences, Laboratory of Molecular and Cellular Neurophysiology, Shanghai 200062, China; 4 Univ. Lyon, Université Claude Bernard Lyon 1, CNRS, CGphiMC UMR5534, 69622 Villeurbanne Cedex, France; florie.reynaud@univ-lyon1.fr (F.R.); julien.falk@univ-lyon1.fr (J.F.); valerie.castellani@univ-lyon1.fr (V.C.); 5 Univ. Lyon, Université Claude Bernard Lyon 1, Inserm, Stem Cell and Brain Research Institute U1208, 69500 Bron, France

**Keywords:** growth factor signaling, NGF, Trk, p75^NTR^, trafficking, lipid rafts, membrane microdomains, caveolin, PC12, CREB, dorsal root ganglion neurons

## Abstract

Membrane microdomains or “lipid rafts” have emerged as essential functional modules of the cell, critical for the regulation of growth factor receptor-mediated responses. Herein we describe the dichotomy between caveolin-1 and caveolin-2, structural and regulatory components of microdomains, in modulating proliferation and differentiation. Caveolin-2 *potentiates* while caveolin-1 *inhibits* nerve growth factor (NGF) signaling and subsequent cell differentiation. Caveolin-2 does not appear to impair NGF receptor trafficking but elicits prolonged and stronger activation of MAPK (mitogen-activated protein kinase), Rsk2 (ribosomal protein S6 kinase 2), and CREB (cAMP response element binding protein). In contrast, caveolin-1 does not alter initiation of the NGF signaling pathway activation; rather, it acts, at least in part, by sequestering the cognate receptors, TrkA and p75^NTR^, at the plasma membrane, together with the phosphorylated form of the downstream effector Rsk2, which ultimately prevents CREB phosphorylation. The non-phosphorylatable caveolin-1 serine 80 mutant (S80V), no longer inhibits TrkA trafficking or subsequent CREB phosphorylation. MC192, a monoclonal antibody towards p75^NTR^ that does not block NGF binding, prevents exit of both NGF receptors (TrkA and p75^NTR^) from lipid rafts. The results presented herein underline the role of caveolin and receptor signaling complex interplay in the context of neuronal development and tumorigenesis.

## 1. Introduction

Membrane microdomains, rich in cholesterol, sphingomyelins, and glycolipids, called lipid rafts, are functional modules of the cell membrane that play a key role in regulating cellular responses to environmental stimuli—e.g., the presence or absence of growth factors or infectious agents—through their capacity to attract or deploy select cellular components [1]. Some domains are characterized by the presence of specific structural and regulatory proteins called caveolins, which, when present in appropriate amounts and with specific post-translational modification, can form caveolae [2,3]. The caveolin family consists of two ubiquitously expressed genes, caveolin-1 (Cav-1) and -2 (Cav-2), and one specifically expressed in smooth and skeletal muscles, caveolin-3 (Cav-3) [4,5]. While expression of Cav-1 or Cav-3 is sufficient for the formation of caveolae, expression of Cav-2 is not [6,7,8].

Cav-1 interacts with numerous proteins involved in signal transduction, which could result in compartmentalization of signaling molecules and their maintenance in an inactive conformation [2,9]. Caveolins influence many diverse physiological processes through their localization in lipid rafts, their role in formation of caveolae, and their specific interaction with both lipid and protein components thereof [10].

At the cell membrane, nerve growth factor receptors TrkA and p75^NTR^ are concentrated in different subtypes of lipid rafts including caveolae [11,12,13]. In addition to its localization in caveolae, the nerve growth factor (NGF)-receptor TrkA was shown to interact with Cav-1 [14]. Consistently, perturbation of caveolae and modification of Cav-1 expression, alters NGF signaling in different cell types [11,14,15]. How caveolins impact NGF signaling from TrkA remains to be clarified. This is particularly interesting considering the different role of Cav-1 and Cav-2 in caveolae formation and their differential regulation during NGF-induced PC12 differentiation [16].

Initially difficult to detect, e.g., [17], caveolins have been observed in brain and several neuronal cell types, playing key roles in neuronal signaling, underlined by correlative and functional observations that certain neurodegenerative diseases may have links to microdomain components including Cav-1 [18,19,20]. Similarly, mutations, loss of, or overexpression of Cav-1 have been associated with numerous cancers [21,22,23,24], resulting in it being considered a bio-marker and potential therapeutic target [25,26,27]. Indeed, Cav-1 is emerging as a key component of circulating exosomes and is elevated, e.g., in melanoma [28] and glioblastoma [29], while its expression in target cells plays a regulatory role in exosome internalization, essential for transmitting molecular information participating in tumor development [30,31].

The present article offers more insight into these issues. Cav-1 overexpression inhibits neurite growth from neurons of the dorsal root ganglia. Cav-1 and Cav-2 differentially modulate NGF signaling events in the PC12 model. Notably, Cav-2 potentiates NGF signaling and the resulting physiological response. By contrast, Cav-1 inhibits NGF signaling without, however, impairing transient MAPK (mitogen-activated protein kinase) pathway activation. Rather, it acts by sequestering NGF receptors and downstream effector, phosphorylated-Rsk2, at the plasma membrane, resulting in the prevention of the phosphorylation of CREB (cAMP response element binding protein). Some insight into the mechanism of action of Cav-1 is afforded by the study of the non-phosphorylatable Cav-1 mutant (S80V), which no longer inhibits TrkA trafficking or CREB phosphorylation. In summary, our results contribute to an understanding of the impact of TrkA and caveolin interplay in tumorigenesis and neuronal differentiation.

## 2. Results

### 2.1. Caveolin-1 (Cav-1) Overexpression Impairs Neurite Growth of Embryonic Sensory Neurons

**Figure 1:**
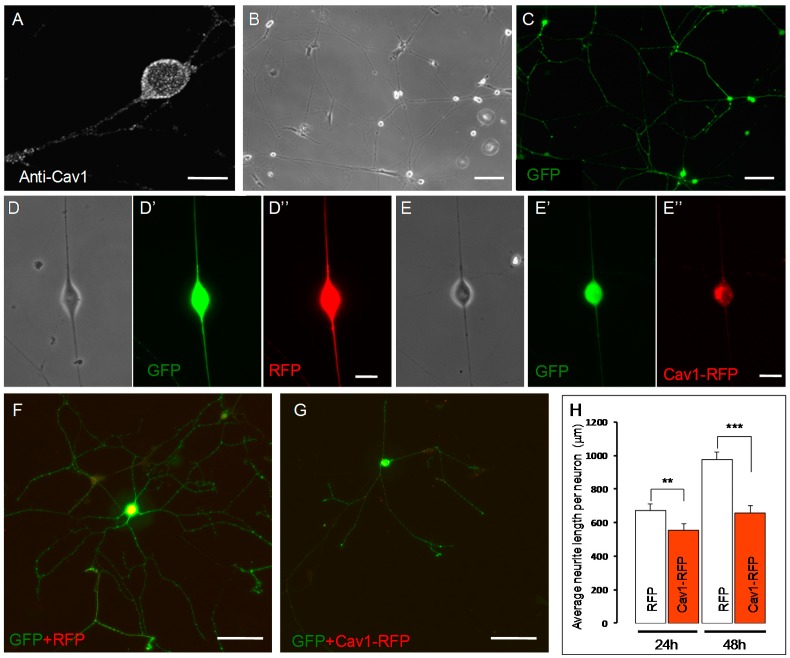
Caveolin-1 (Cav-1) expression inhibits neurite outgrowth from mouse Dorsal root ganglia (DRG) neurons in culture. Cav-1 is detected in both the soma and the neuritic processes of E14.5 DRG neurons (**A**); Neon^®^ transfection leads to efficient electroporation of E14.5 DRG neurons with little adverse effects (**B**,**C**); Neurons co-expressing GFP (Green Fluorescent Protein) and RFP (Red Fluorescent Protein) (**D–D’’**) or GFP and Cav-1-RFP (**E–E”**) can differentiate in vitro. Phase images (**D** and **E**) exemplify the morphology of GFP (**D’** and **E’**) and RFP (**D”**) or Cav1-RFP (**E”**) expressing neurons. Nevertheless, neurons expressing Cav-1-RFP grew shorter processes than neurons expressing RFP (**F**,**G**); The length of GFP positive neurites measured and divided by the number of transfected neurons (**H**); Results are pooled from three sets of cultures, each culture included four mosaic fields containing >250 transfected cells. Mean ± SEM; (** *p* < 0.01; *** *p* < 0.00001). Statistical analysis was performed using the two-tail paired Student’s *t*-test. Scale bars represent 100 µm in **B**, **C**, **F** and **G,** and 10 µm in **A**, **D”** and **E’’**.

Sensory neurons from dorsal root ganglia (DRG) express a combination of Trk neurotrophin receptors during development, whereas in adult, the different receptors are mainly expressed individually in discrete sub-populations [32]. NGF was shown to regulate morphology and growth of DRG axons in vitro [33,34,35]. Expression studies were performed in embryonic DRG neurons from stage E14.5 cultured on laminin substratum. At this stage, TrkA is expressed in nearly 80% of the DRG neurons and NGF was shown to enhance laminin-induced outgrowth [35,36]. Consistent with previous reports in rat neurons, we found that Cav-1 is expressed by TrkA-positive DRG neurons and is found both in the soma and the neurites [16] (Figure 1A). To get efficient transfection with low amounts of cells, electroporation of DRG neurons was optimized using the Neon^®^ transfection system [37]. Using this procedure, we transfected more than 50% of the neurons (Figure 1B,C). We found that many DRG neurons transfected with Cav-1-RFP (red fluorescent protein) [38] and GFP (green fluorescent protein) differentiate (Figure 1D–G. Quantification of the total neurite length of transfected neurons showed that neurons co-expressing Cav-1-RFP and GFP grew shorter neurites than RFP- and GFP-expressing ones. Indeed, average length decreased by 18% in Cav-1 overexpressing neurons after one day in vitro (12 montages representative of 339 RFP-positive neurons and 293 Cav-1-RFP-expressing neurons, *p* = 0.018). In parallel with a progressive accumulation of Cav-1, this decrease reached 33% after two days (434 RFP-positive and 308 RFP-positive neurons, *p* = 0.00009) (Figure 1H). This effect was even stronger when the amount of plasmid is doubled, further supporting a dose-dependent effect. Average total neurite length dropped by 34% (361 RFP-positive and 416 RFP-positive neurons, *p* = 0.002) after the first day and 41% on the second day (296 RFP-positive and 351 RFP-positive neurons, *p* = 0.000002). Thus, overexpression of Cav-1 appears to impair neurite growth of DRG neurons as it does in PC12 cells [14,16]. The PC12 line was used in the subsequent studies.

### 2.2. Effect of Cav-1 and Cav-2 on Nerve Growth Factor (NGF)-Induced Neurite Formation in PC12 Cells

Interestingly, Cav-1 and Cav-2 exhibit different kinetics of expression during NGF-induced differentiation. In agreement with previously-published studies [16], Cav-1 expression is almost undetectable in sparse populations and progressively increases in PC12 cells in response to NGF. In contrast, Cav-2 expression increases to a maximum after two days of treatment with NGF, then drops precipitously (Figure S1). Since, Cav-1 and Cav-2 have an expression peak during late and early phases of neuritogenesis, respectively, we wondered whether they could have different effects on NGF-induced PC12 cell morphological differentiation.

**Figure 2:**
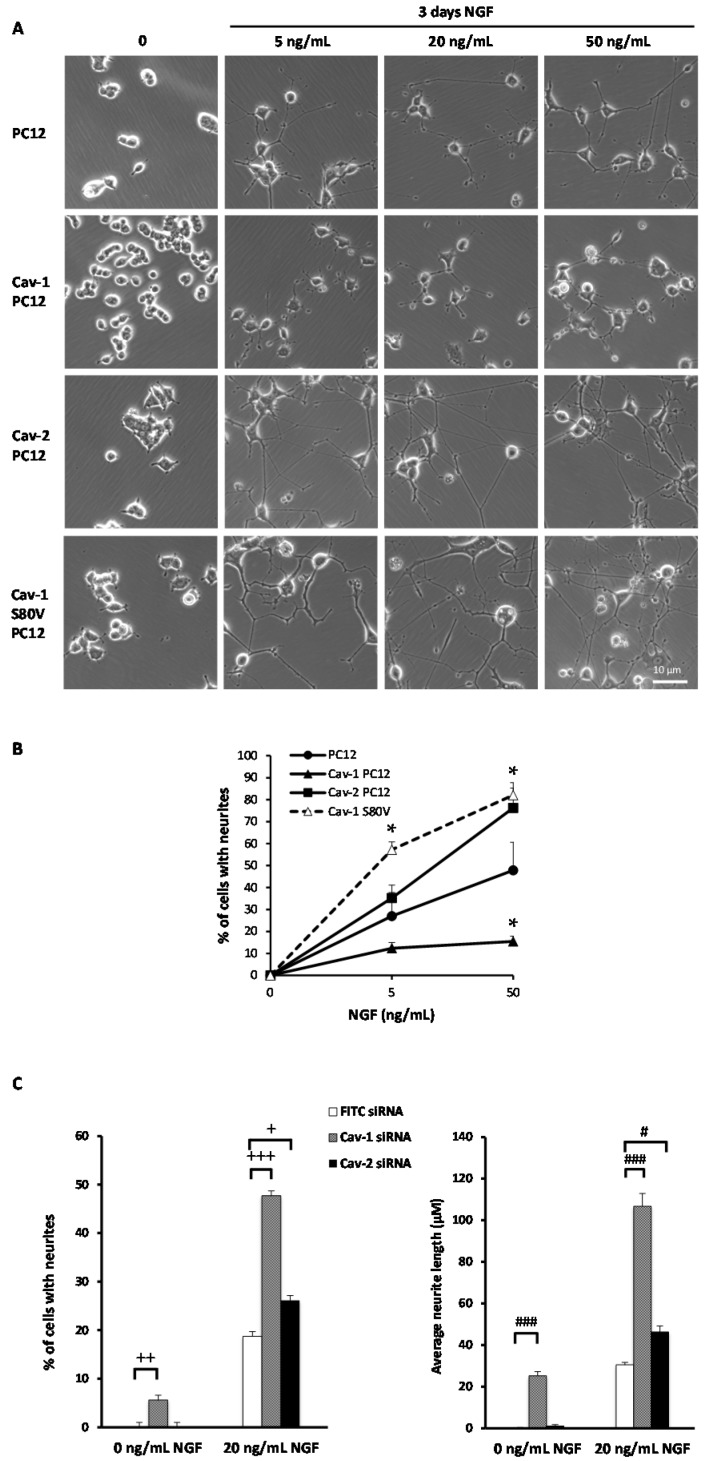
Effect of Cav-1 and Cav-2 expression on NGF-induced PC12 cell differentiation. (**A**) Normal PC12 cells, stably-transfected Cav-1 PC12 cells Cav-2-PC12 or Cav-1 S80V PC12 cells were plated at a density of 10^5^ cells/well on collagen/poly-lysine coated six-well plates. Cells were maintained in DMEM (Dulbecco’s Modified Eagle’s medium) supplemented with serum for 24 h After 17 h of serum deprivation, NGF was added at increasing concentrations (0, 5, 20, 50 ng/mL NGF). After three days in culture, cells were photographed; (**B**) Analysis of neurite outgrowth. Quantification of the percentage of cells exhibiting neurites longer than three cell body diameters after 72 h of NGF treatment at 0, 5, and 50 ng/mL. Approximately 100 cells per condition from five independent experiments were taken into account. Values are mean percentage ± SEM. Statistical significance of the observations between PC12 cells and Cav mutant (Cav-1; Cav-2; Cav-1 S80V) PC12 cells are indicated by * *p* < 0.05 (unpaired, two-tail Student’s *t*-test). (**C**) Effect of siRNA towards Cav-1 and Cav-2 on neurite outgrowth. Normal PC12 cells, plated on collagen/poly-lysine coated 24-well plates (2 × 10^4^ cells per well), were transiently transfected with 30 nM siRNA against caveolin-1; caveolin-2 and FITC (fluoresceine isothiocyanate) conjugated scrambled siRNA. Cells were maintained in DMEM with 0 or 20 ng/mL NGF. After 3 days, cells were photographed and neurite outgrowth was quantified by ImageJ (Average 400 cells per condition compiled from two independent experiments. Values are mean ± SEM. Statistical significance of the observations between PC12 cells and Cav-expressing (Cav-1; Cav-2) PC12 cells are indicated by + *p* < 0.005; ++ *p* < 0.0005; +++ *p* < 0.00005 (% cells with neurites) and # *p* < 0.0005; ### *p* < 0.0000001 (Average neurite length) as ascertained by the unpaired, two-tail Student’s *t*-test).

In order to evaluate the impact of caveolin expression on global NGF signaling, normal PC12 cells were stably-transfected and assayed for transgene expression. Clones expressing Cav-1 or Cav-2 were selected for subsequent studies with most experiments being performed on two or more clones (Table S1). NGF induces PC12 cells to stop proliferating and to differentiate into a sympathetic neuron-like phenotype [39]. Normal PC12 cells, Cav-1 PC12 cells and Cav-2 PC12 cells were plated at low density and exposed for three days to NGF concentrations ranging from 5 to 50 ng/mL. In response to NGF, normal PC12 cells extended a characteristic neurite network in a dose-dependent manner whereas Cav-1 PC12 cells did not elicit any significant process extension at any of the NGF concentrations evaluated (Figure 2A), a result consistent with previous studies [14,16]. In contrast, Cav-2 PC12 cells exposed to NGF developed a more extensive and denser neurite network than normal PC12 cells (Figure 2A). NGF-induced morphological differentiation of Cav-2 PC12 cells thus appears to be potentiated in comparison to the response of normal PC12 cells. The global evaluation of differentiation of each cell line at the different NGF concentrations is represented in Figure 2B. These observations suggest that Cav-1 and Cav-2 have opposite effects on NGF-induced PC12 cell morphological differentiation. Corroborating this observation, exposure of the PC12 cells to siRNA towards Cav-1 resulted in an increase of differentiation while siRNA to Cav-2 would appear to have had a minimal effect (Figure 2C). The extremely low levels of expression of Cav-1 and Cav-2 in exponentially growing PC12 cells made it difficult to appreciate the relative impact of the two siRNA on the expression of their respective targets. These results are therefore to be taken with the necessary caution.

### 2.3. Effect of Cav-1 and Cav-2 on the Anti-Mitogenic Response to NGF

**Figure 3:**
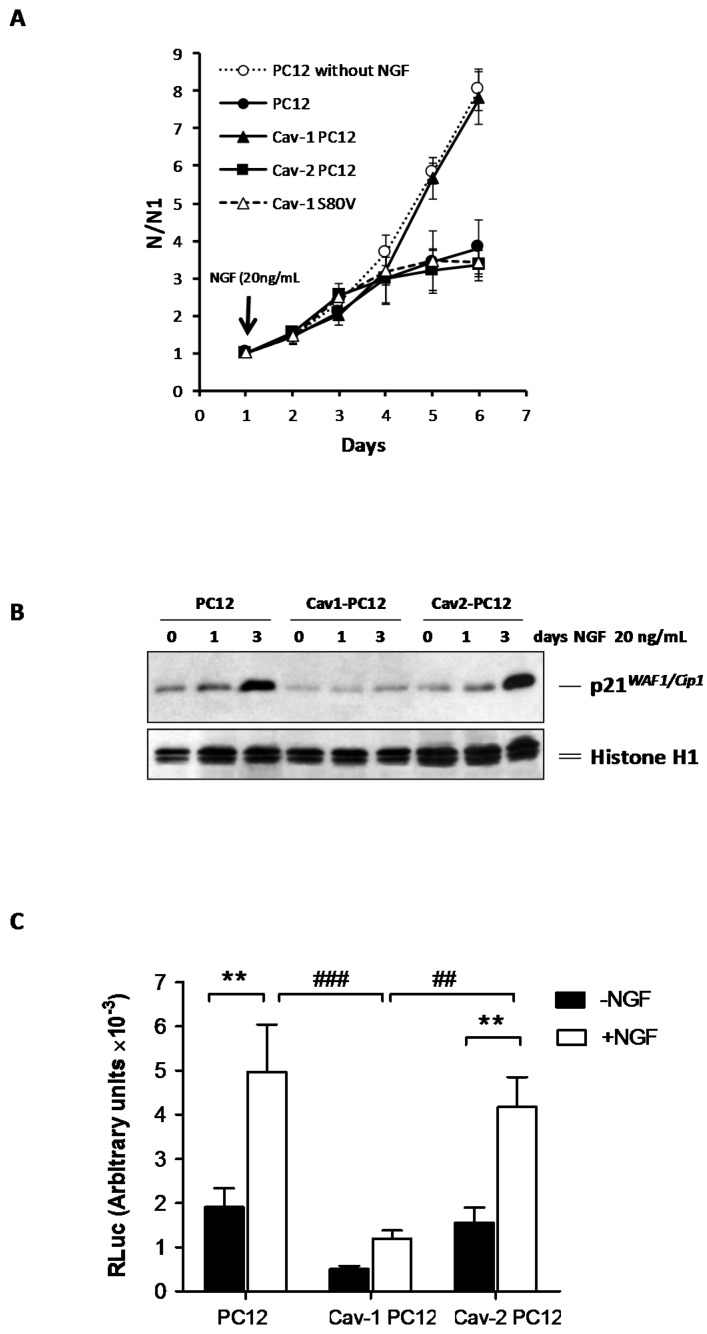
Effect of Cav-1 and Cav-2 expression on the anti-mitogenic effect of NGF. (**A**) Normal PC12 cells, Cav-1 PC12 cells, Cav-2 PC12, and Cav-1 S80V PC12 cells were plated at a low density in 25 cm^2^ dishes containing a medium supplemented with serum. NGF 20 ng/mL was added to the medium on day 2, except for one series of dishes that was left untreated. Cell number was counted every day for six days. The experiment was performed three times, with two different clones of Cav-1 PC12 and Cav-2-PC12 cells. Cell number is normalized to cell number at day 1 (N1). Values are mean ± SEM; (**B**) Normal PC12 cells, Cav-1 PC12 cells, and Cav-2 PC12 cells were exposed to 20 ng/mL NGF for 0, 1, or 3 days. Proteins were extracted and resolved by SDS-PAGE and immunoblotted with CP36 monoclonal antibody directed against p21^WAF1/Cip1^. Equal loading was verified by reprobing the same blot with anti-Histone H1 antibody; (**C**) The NGF-dependent induction of p21^WAF1/Cip1^ was analyzed in normal PC12 cells, Cav-1 PC12 cells, and Cav-2 PC12 cells. Cells were transiently transfected with the minimal p21 promoter–Luciferase reporter (p21–Luc) and treated or not with NGF (50 ng/mL for 48 h) as described in the Materials and methods section (Mean ± SEM of three independent experiments. Statistical significance was determined using a regular two-way ANOVA test with Bonferroni *post hoc* tests ** *p* < 0.01 versus the corresponding–NGF group. ## *p* < 0.01, ### *p* < 0.001 versus the Cav-1 PC12 + NGF group).

PC12 cells respond to NGF by induction of an anti-mitogenic response [39] elicited by cell cycle arrest in the G1 phase [40], and the triggering of differentiation. Normal PC12 cells, Cav-1 PC12 cells and Cav-2 PC12 cells were plated at low density in the presence of serum and grown for two days prior the addition of 20 ng/mL NGF. In the presence of NGF, normal PC12 cells and Cav-2 PC12 cells undergo a final round of division and stop proliferating with similar kinetics (Figure 3A). It would appear that the potentiation of NGF-induced differentiation, observed with Cav-2 overexpression, is not a consequence of an enhanced anti-mitogenic response. In contrast, Cav-1 PC12 cells did not stop proliferating after NGF addition and showed a growth rate similar to that of exponentially-growing normal PC12 cells in absence of NGF (Figure 3A). The anti-mitogenic effect of NGF is propagated via the induction of the cyclin-dependent kinase inhibitor p21^WAF/Cip1^ [41,42]. To test if Cav-1 or Cav-2 expression alters p21^WAF/Cip1^ levels, equal numbers of cells exposed to 20 ng/mL of NGF for 0, 1, or 3 days were lysed and protein expression ascertained by western analysis. Figure 3B shows that p21^WAF/Cip1^ levels in normal PC12 cells and in Cav-2 PC12 cells markedly increase during the time course of NGF treatment. No change in p21^WAF/Cip1^ level was detectable in Cav-1 PC12 cells exposed to NGF.

The minimal p21^WAF/Cip1^–promoter luciferase construct (p2193S-Luc) was used as reporter gene for the NGF signaling pathway [43,44,45]. In normal PC12 cells treated with NGF for 48 h, the promoter is activated as ascertained by an increase in firefly luciferase activity. This activation of the p21 promoter is also found in Cav-2 PC12 cells. In contrast, NGF-induced p21 promoter activation is reduced in Cav-1 PC12 cells (Figure 3C).

These results indicate that Cav-1, but not Cav-2 expression results in inhibition of the anti-mitogenic effect of NGF, at least in part, by impairing activation of transcription of p21^WAF/Cip1^.

### 2.4. Effect of Cav-1 and Cav-2 on NGF-Induced TrkA and p75^NTR^ Internalization

**Figure 4:**
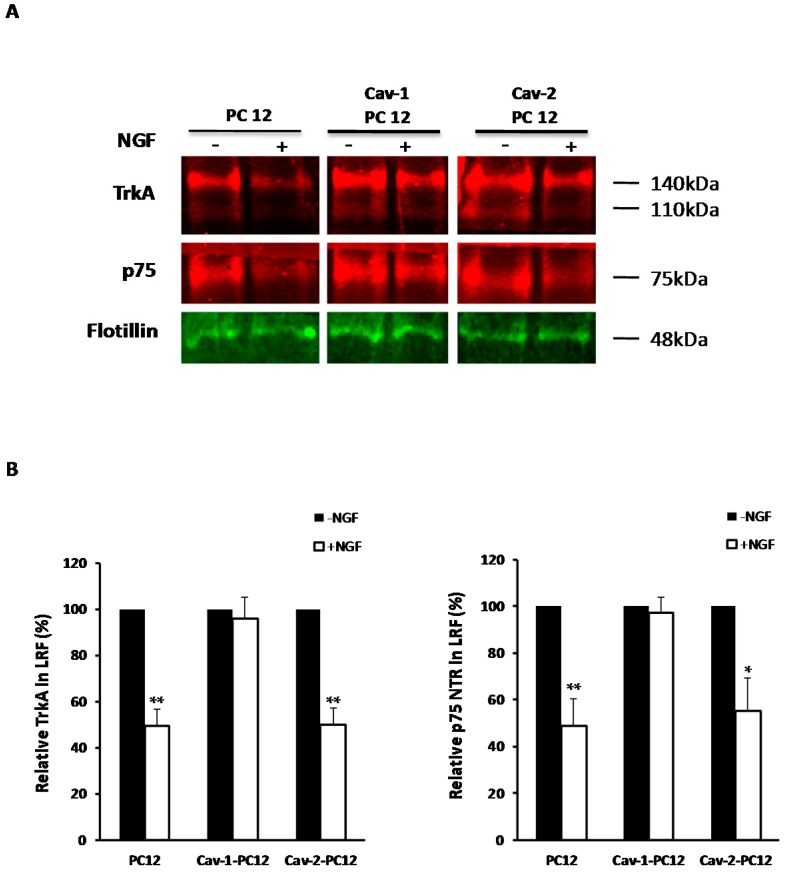
Effect of Cav-1 and Cav-2 expression on NGF receptor exit from lipid rafts. (**A**) TrkA and p75^NTR^ levels in the lipid raft fraction (LRF) before and after addition of NGF (20 ng/mL for 45 min) to cultures of normal PC12, Cav-1 PC12, and Cav-2-PC12 cells isolated, as described in the Materials and Methods. Lipid raft fractions were then subjected to Western analysis. Nitrocellulose membranes were probed with RTA, anti-p75^NTR^ and anti-flotillin-1 antibodies. Flotillin-1 was used as a loading control. The Odyssey imaging system was used for quantitative infrared fluorescence detection of the relative amount of proteins; (**B**) Analysis of TrkA and p75^NTR^ exit from lipid rafts. Level of TrkA and p75^NTR^ in lipid rafts was normalized for flotillin-1 level for each sample. Systematic comparison of data with and without flotillin correction gave identical results. TrkA and p75^NTR^ exit from the lipid raft fraction was then analyzed and represented as percent of the TrkA in lipid rafts compared to the amount observed in the absence of NGF, considered as 100 percent. (Mean ± SEM of three independent experiments. Statistical significance of the effect of addition of NGF vs. the absence of NGF, was ascertained using the unpaired Student’s *t*-Test. * *p* < 0.05, ** *p* < 0.01.)

NGF receptor trafficking is essential for regulating many of the subsequent cellular responses [13,46,47,48,49,50,51,52,53,54,55]. The effect of Cav-1 and Cav-2 expression on TrkA was monitored in clones of PC12 cells stably expressing these proteins. Following NGF treatment, Figure 4A shows that TrkA and p75^NTR^ exit from lipid rafts in normal PC12 and Cav-2 PC12 cells. In contrast, TrkA and p75^NTR^ remain in lipid rafts in Cav-1 expressing cells, indicating that Cav-1 is retaining NGF receptors in lipid rafts. Quantification of several independent experiments (Figure 4B) shows that Cav-1 almost totally inhibits the exit of TrkA and p75^NTR^ from lipid rafts, whereas Cav-2 does not.

**Figure 5:**
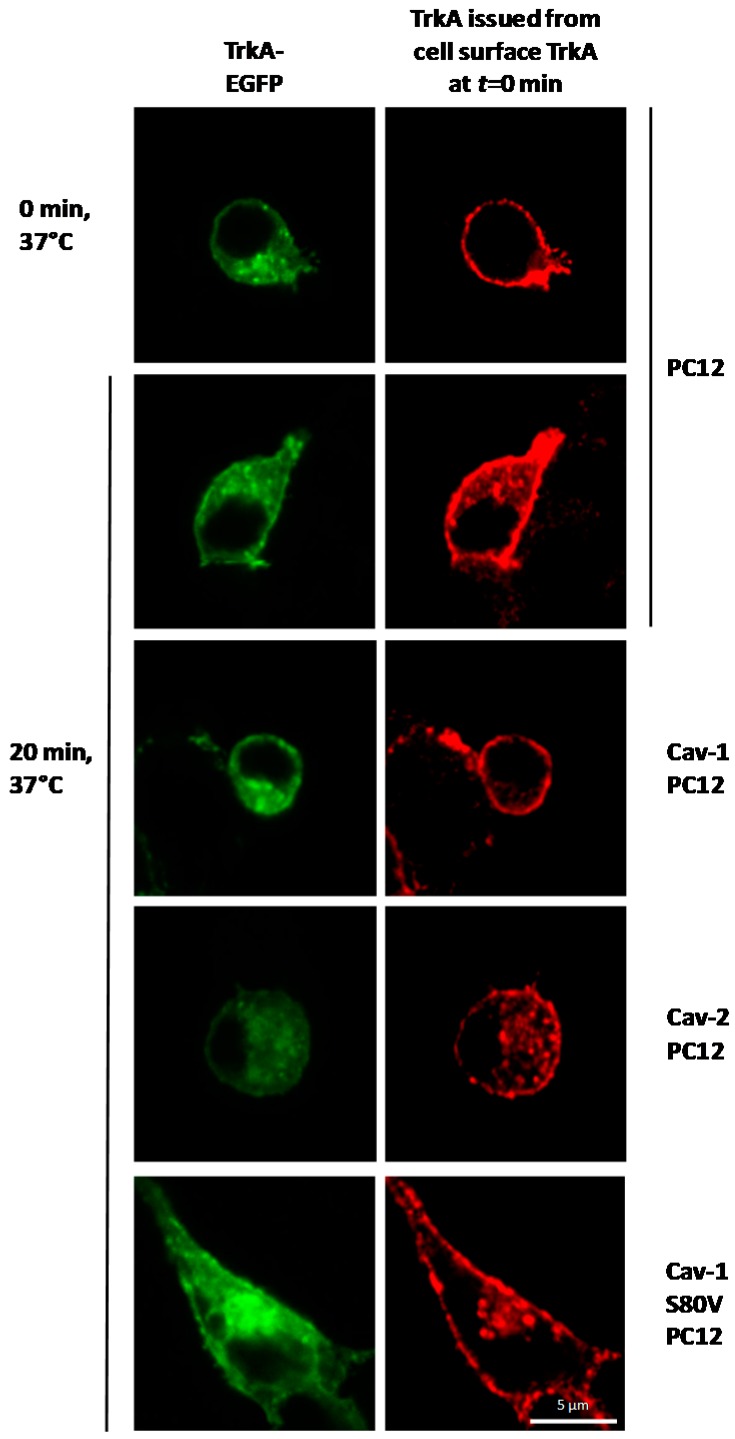
Effect of Cav-1 and Cav-2 expression on TrkA trafficking. Internalization of TrkA was provoked by RTA addition to normal PC12, Cav-1 PC12, Cav-2 PC12, and Cav-1 S80V PC12 cells transiently expressing TrkA–EGFP chimerae (green), as described in the Materials and Methods. After fixation and permeabilization of the cell, RTA localization was determined using a secondary antibody labeled with rhodamine (red) at the indicated times. Rhodamine fluorescence indicates the location of TrkA–RTA and TrkA–EGFP–RTA complexes that were at the cell surface at the beginning of the experiment (Cell Surface TrkA at *t* = 0 min). Distribution of cell surface TrkA (at *t* = 0) in Cav-1 PC12, Cav-2-PC12, and Cav-1 S80V PC12 cells after 0 min at 37 °C is identical to that observed in PC12 cells.

Ligand-induced internalization of TrkA and subcellular localization of TrkA were evaluated using confocal microscopy (Figure 5). Normal PC12 cells, Cav-1 PC12 cells and Cav-2 PC12 cells were transiently transfected with a chimeric TrkA-EGFP receptor [55]. Expression of this receptor, together with the use of RTA antibody as an NGF agonist [55,56] allow simultaneous monitoring of two different pools of TrkA in a single cell. (i) “TrkA-EGFP” reflects EGFP fluorescence, which corresponds to all of the cellular localizations of TrkA-EGFP; (ii) “Cell surface TrkA at *t* = 0 min” is identified by incubation of cells at 4 °C for 30 min in presence of RTA, followed by fixation, permeabilization and labeling of cells with rhodamine-conjugated anti-rabbit antibody. This allows the detection of TrkA-RTA complexes, which are initially at the cell surface. Following the shift to 37 °C, it is possible to monitor the fate of the receptors that have moved from the cell surface to intracellular locations during the incubation time. As shown in Figure 5, first row, incubation of PC12 cells in the presence of RTA at 4 °C does not allow receptor internalization, reflected by the presence of RTA-TrkA complexes exclusively at the cell surface. Incubation of PC12 cells and of Cav-2 PC12 cells for 20 min at 37 °C in the presence of RTA leads to partial redistribution of the TrkA–antibody complex inside the cells. Intracellular RTA–TrkA complexes correspond to internalized receptors. By contrast, following the shift to 37 °C of Cav-1 PC12 cells treated with RTA, little or no intracellular RTA-TrkA complexes are detected, indicating that TrkA internalization is impaired in Cav-1 overexpressing cells.

Thus, the differential internalization of TrkA in the presence of increased levels of Cav-1 and Cav-2 appears to mirror the functional outcome of Cav-1 and Cav-2 overexpression on PC12 differentiation.

### 2.5. Interplay of p75^NTR^ and TrkA

**Figure 6:**
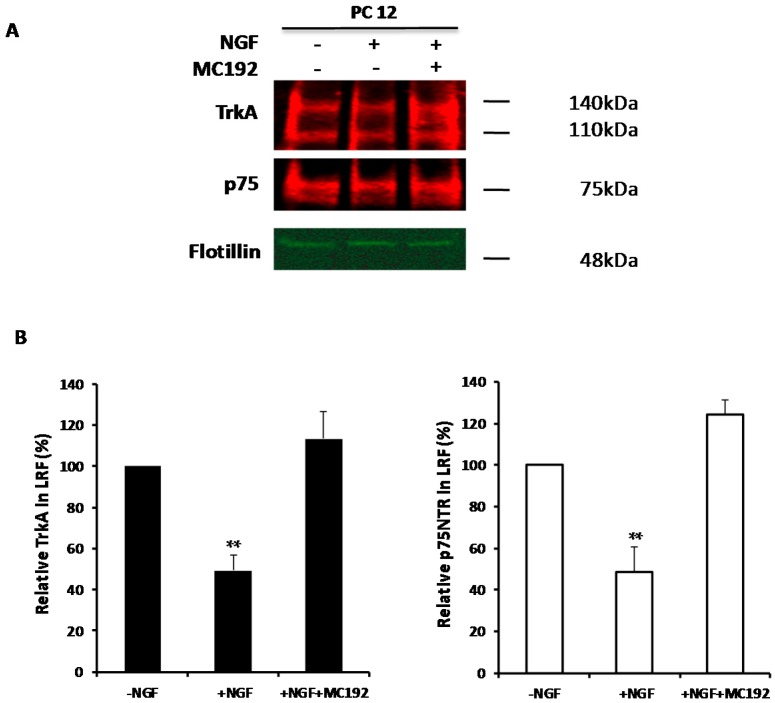
Effect of MC192 on TrkA exit from lipid rafts before and after addition of NGF. (**A**) Lipid rafts were isolated from normal PC12 cells treated with NGF (20 ng/mL for 45 min), treated with p75^NTR^ MC192 monoclonal antibody (8 ng/mL) for 30 min prior to and during NGF exposure (20 ng/mL for 45 min) and from untreated normal PC12 cells were isolated as described in the Materials and Methods. Lipid rafts were then subjected to Western analysis. Nitrocellulose membranes were probed with RTA and anti-flotillin-1 antibody to detect TrkA expression and flotillin-1 was used as a loading control. Revelation was achieved using quantitative infrared fluorescence detection using the Odyssey imaging system; (**B**) Analysis of TrkA exit from lipid rafts. Level of TrkA in the lipid rafts was normalized to the Flotillin-1 level for each sample. TrkA exit from the lipid raft fraction (LRF) was then analyzed and represented as percent of the TrkA in lipid rafts vs. TrkA in normal PC12 cells considered as 100%. (Mean ± SEM of four independent experiments. Statistical significance vs. cells in the absence of NGF, was ascertained using the unpaired, two-tail Student’s *t*-Test. ** *p* < 0.01.)

When PC12 cells are treated with MC192 monoclonal antibody, specific for the extracellular domain of p75^NTR^ [57], TrkA internalization is inhibited, even in presence of high NGF concentrations [58]. Figure 6A (characteristic Western Blot) and Figure 6B (quantitation of multiple experiments) illustrate that exposure of PC12 cells to this anti-p75^NTR^ antibody almost completely abolishes NGF-induced TrkA and p75^NTR^ exit from lipid rafts, much like Cav-1 expression. This suggests that part of the role of p75^NTR^ in TrkA internalization may be to participate in, or regulate, the exit from lipid rafts in response to NGF.

### 2.6. Effect of Cav-1 and Cav-2 on Activation of TrkA and Downstream Signaling Effectors

**Figure 7:**
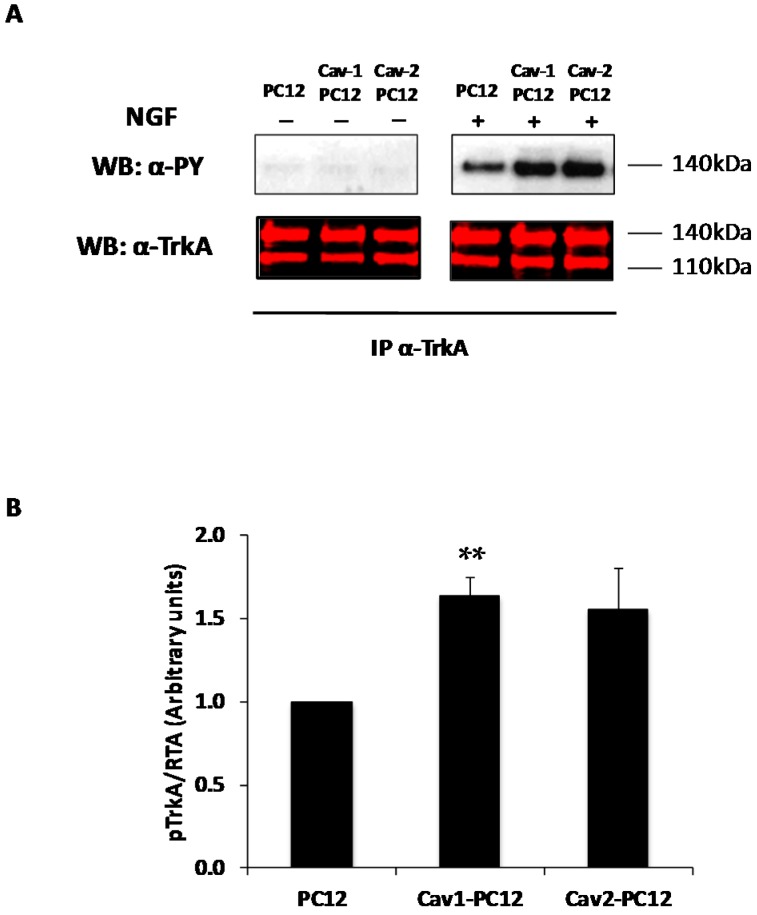
Effect of Cav-1 and Cav-2 expression on TrkA activation. Normal PC12 cells, Cav-1 PC12 cells and Cav-2 PC12 cells were left untreated or treated with NGF 20 ng/mL for 10 min after 17 h of serum deprivation. (**A**) One mg protein of cell lysate was immunoprecipitated with a polyclonal antibody directed against TrkA C-terminal domain (anti-Trk C-14), and immunoprecipitates were subjected to analysis by Western Blot (WB). Membrane was probed with an antibody to phosphotyrosine (α-PY) followed by a secondary antibody conjugated to horseradish peroxidase that allows chemiluminescence detection; The same blot was re-probed with RTA polyclonal antibody to TrkA followed by a secondary antibody conjugated to a fluorochrome (**B**), which allows infrared fluorescence detection using the Odyssey imaging system without cross-reacting with the first antibodies (Mean ± SEM from three independent experiments. Statistical significance vs. PC12 cells was ascertained using the unpaired, two-tail Student’s *t*-Test. ** *p* < 0.01).

NGF signaling and PC12 cell differentiation are mediated via TrkA activation [59]. Since receptor endocytosis is important to orchestrate NGF signaling, we tested whether Cav-1 and Cav-2 differentially regulate the canonical NGF signaling cascade via Ras/Raf/MAPK. Normal PC12 cells, Cav-1 PC12 cells and Cav-2 PC12 cells were exposed to 20 ng/mL NGF for 10 min. In the absence of NGF, no TrkA phosphorylation was detectable in any cell line. However, 10 min exposure to NGF resulted in marked tyrosine phosphorylation in all cell lines, with an increased phosphorylation of TrkA in Cav-1 and Cav-2 PC12 cells compared to TrkA phosphorylation in normal PC12 cells (Figure 7A (characteristic western blot) and Figure 7B (quantitation of multiple experiments).

Thus, Cav-1 expression does not appear to inhibit phosphorylation of TrkA under the experimental conditions used herein. To further characterize the impact of Cav-1 and Cav-2, downstream effectors of the NGF signaling pathway were monitored. Phosphorylated TrkA leads to MAPK activation that induces Rsk phosphorylation. Rsk will ultimately phosphorylate CREB. Normal PC12 cells, Cav-1 PC12 cells, and Cav-2 PC12 cells were treated with 20 ng/mL NGF for 0 to 6 h. The activation status of several effectors of the Ras-MAPK pathway was analyzed by Western blot using antibodies directed towards their phosphorylated forms.

**Figure 8:**
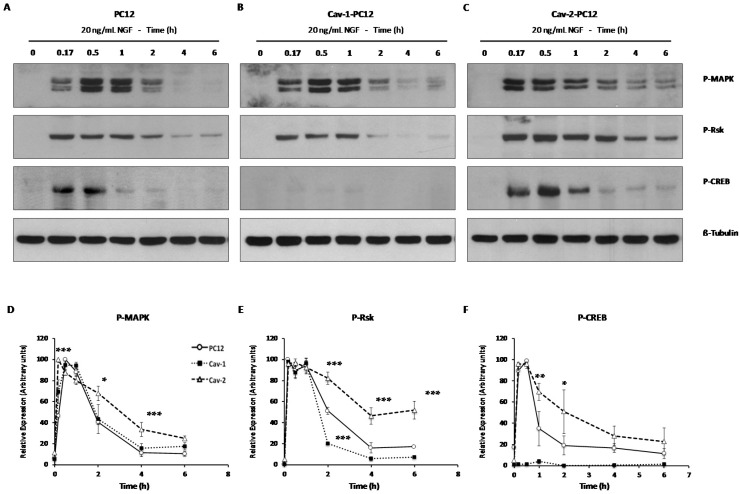
Kinetics of activation of the MAPK pathway in PC12 cells stably-transfected with Cav-1 or Cav-2. Normal PC12 cells (**A**); Cav-1 PC12 cells (**B**) and Cav-2 PC12 cells (**C**) were exposed for up to 6 h to NGF 20 ng/mL. Cells were collected and proteins were extracted in lysis buffer. Protein concentrations in the lysates were determined and 40 µg were used for Western blot analysis. Blots were probed with phospho-specific antibodies to MAPK, Rsk2, and CREB. Equal loading was controlled using a β-tubulin antibody; (**D**–**F**) Scans of gels were quantified using the gel analysis function of ImageJ software and analyzed as described in the Materials and Methods section; (**D**) P-MAPK; (**E**) P-Rsk; (**F**) P-CREB. In all, PC12 cells: Solid line with open circles; Cav-1-PC12 cells: Dotted line with filled squares; Cav-2-PC12: Dashed line with open triangles. Results are presented as the % maximal response in order to be able to compare multiple experiments. (Mean ± SEM derived from 24 gels generated in nine independent experiments performed on Cav-1 clones 3, 12, 16 and Cav-2 clones 5, 11, and 23 (see Supplementary Figure S2) having similar relative expression levels. Statistical significance was ascertained using the two-way ANOVA test with Bonferroni *post hoc* tests. *p* values are shown for results obtained with caveolin clones vs. those obtained with PC12. * *p* < 0.01, ** *p* < 0.005, *** *p* < 0.0005).

NGF treatment of normal PC12 cells results in a prolonged activation of MAP kinases ERK1 and ERK2 (extracellular signal-regulated kinase 1 & 2), with phosphorylation peaking at 30 min treatment, and detectable up to 2 h after NGF addition (Figure 8A). Rsk2 phosphorylation is also readily detected and lasts up to 6 h after addition of NGF, while CREB phosphorylation on serine 133 peaks at 30 min, decreasing to the limit of detection after 2 h.

Cav-2 PC12 cells exhibit an even longer duration of MAPK, Rsk2, and CREB activation. The phosphorylated forms of the proteins are easily detectable up to 6 h after NGF addition (Figure 8C). These results show that TrkA activation in Cav-2 PC12 cells is accompanied by the lengthening of the duration of MAPK pathway activation. Extended MAPK activation has been proposed to be critical in mediating the differentiation effects of NGF in PC12 cells [60], this could provide an explanation for the potentiating effects of Cav-2 expression on NGF-induced differentiation.

In Cav-1 PC12 cells, Erk1 and Erk2 are activated with kinetics very similar to that observed in normal PC12 cells (Figure 8B). RSK2 is also activated, although phosphorylation does not last as long as that observed in normal PC12 cells. CREB phosphorylation on Serine 133 was barely detectable. This result shows that the activation of CREB is inhibited in Cav-1 PC12 cells. Thus, it appears that Cav-1 expression blocks CREB activation while Cav-2 expression sustains it.

### 2.7. Effect of Cav-1 and Cav-2 Expression on TrkA Effector Localization

**Figure 9:**
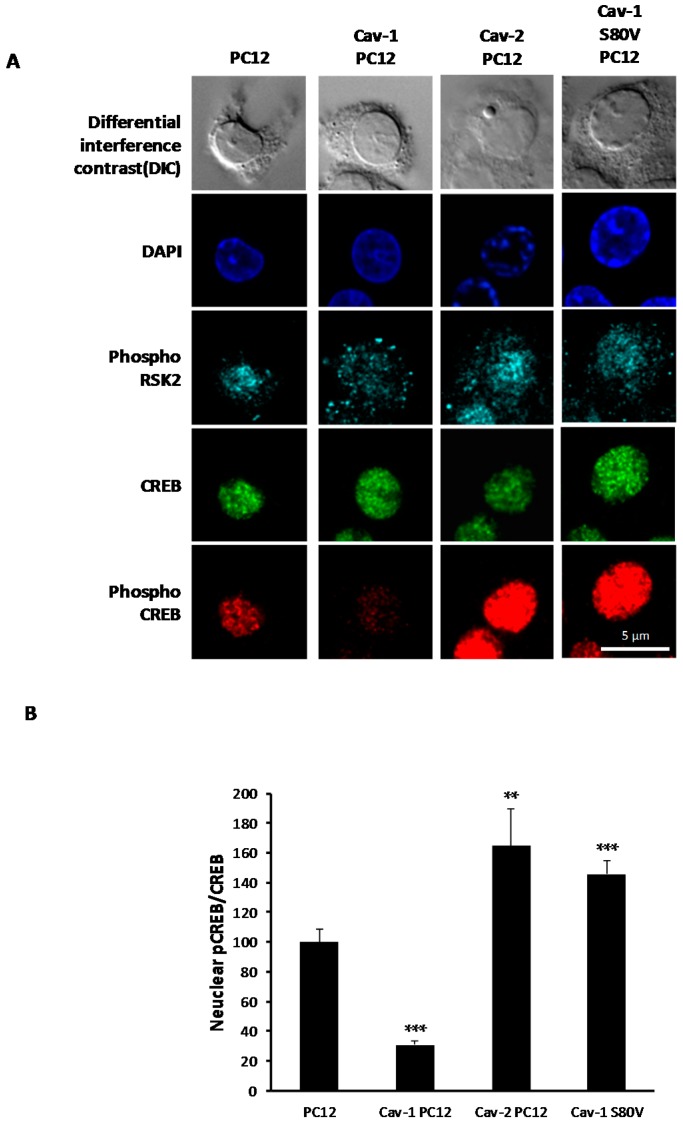
Effect of Cav-1, Cav-2 and Cav-1 S80V expression on TrkA effector localization. (**A**) Normal PC12 cells, Cav-1 PC12 cells Cav-2 PC12, and Cav-1 S80V PC12 cells were plated on collagen/poly-lysine coated coverslips and exposed to 20 ng/mL NGF for 30 min after 17 h of serum deprivation. Cells were fixed and simultaneously stained with an anti-pRsk2 antibody (cyan), an anti-CREB antibody (green) and an anti-pCREB antibody (red). Cells were mounted in mounting medium containing DAPI to visualize the nucleus; (**B**) Quantification of CREB phosphorylation level in the nucleus. For each single cell, quantitation of fluorescence representative of pCREB and CREB in the nucleus was assessed using ImageJ software. Level of CREB phosphorylation was evaluated by dividing pCREB fluorescence by CREB fluorescence. Results are represented as percent of CREB phosphorylation observed with CREB phosphorylation in normal PC12 cells considered as 100 percent. (Mean of 20 to 70 cells per conditions ± SEM. Statistical significance vs. PC12 cells, was ascertained using the unpaired, two-tail Student’s *t*-Test. ** *p* < 0.01; *** *p* < 0.001.)

Normal activation of CREB in response to NGF is achieved via phosphorylation of serine 133 by Rsk2 [61,62]. Following activation, pRsk2 is translocated into the nucleus where it phosphorylates CREB [62]. Since Rsk2 is phosphorylated in Cav-1 overexpressing PC12, we investigated if the lack of CREB phosphorylation was due to impaired pRsk translocation. Subcellular localization of pRsk2, CREB and pCREB was studied by confocal immunofluorescence microscopy in the caveolin-expressing cells. Figure 9A,B show that pCREB was detected in the nucleus of normal PC12 cells and Cav-2 PC12 cells with a greater level of activation in Cav-2 PC12 cells. In the nucleus of Cav-1 PC12 cells, the level of CREB activation was essentially completely inhibited. Furthermore, in normal PC12 cells and in Cav-2 PC12 cells exposed to 20 ng/mL NGF for 30 min, pRsk2 is mainly located in the nucleus. By contrast, in Cav-1 PC12 cells exposed to NGF, the level of pRsk2 in the nuclear compartment is drastically diminished. Moreover, accumulation of pRsk2 can be seen in isolated foci at the periphery of the cell. Thus the absence of enhanced nuclear translocation of pRsk2 in Cav-1 PC12 cells may explain why CREB is not phosphorylated in the nucleus of these cells.

**Figure 10:**
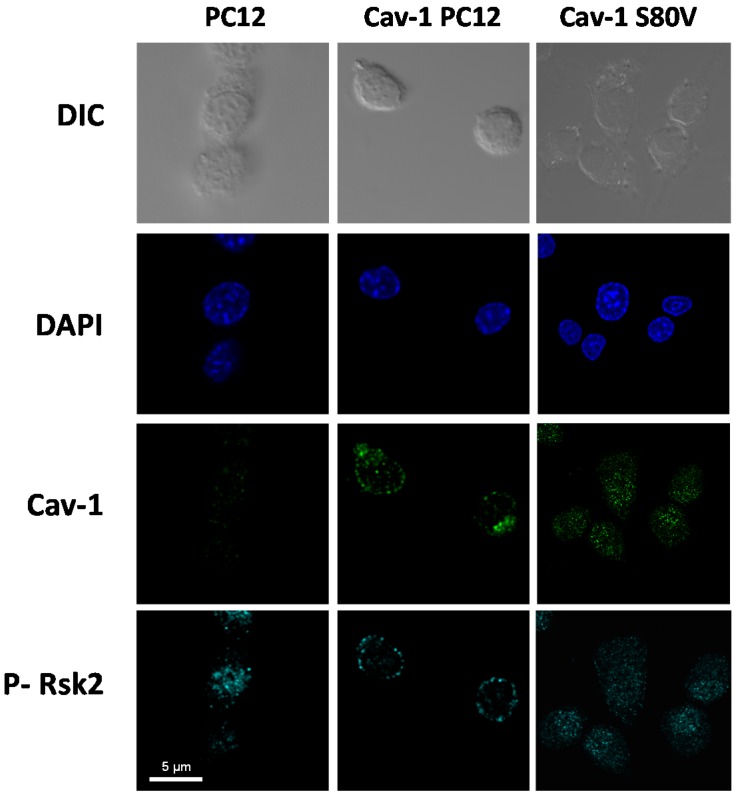
Cav-1, Cav-1 S80V, and phospo-Rsk2 localization in normal, Cav-1 and Cav-1 S80V PC12 cells. Normal PC12 cells, Cav-1, and Cav-1 S80V PC12 cells were plated on collagen/poly-lysine coated coverslips and exposed to 20 ng/mL NGF for 30 min after 17 h of serum deprivation. Cells were fixed and simultaneously stained with an anti-pRsk2 antibody (cyan) and an anti-Cav-1 antibody (green).

Figure 10 shows the cytolocalization of Cav-1, Cav-1 S80V and pRSK2 after 30 min exposure of normal, Cav-1 and Cav-1 S80V PC12 cells to 20 ng/mL NGF. Cav-1 was predominantly located at the periphery of the Cav-1 PC12 cells, whereas Cav-1 S80V was essentially located intracellularly.

### 2.8. Effect of Cav-1 Point Mutations on the NGF Signaling Pathway

Paracrine/autocrine activation of NGF signaling has been implicated in tumor progression, cell survival and metastasis and deletion or mutation of Cav-1 have been reported for certain cancers, notably breast cancer [63,64,65,66,67] while overexpression has been observed in certain prostate cancer lines [26,68]. Mutations of specific codons, e.g., those coding for Pro132 and Ser 80, have been reported to potentially override or inactivate the growth inhibitory activity of Cav-1 [24,69,70].

PC12 cells were stably-transfected with Cav-1 S80V (non-phosphorylatable form) and assayed for transgene expression. Individual clones expressing Cav-1 S80V were selected, and the impact on specific parameters evaluated as described above with cells expressing wild-type caveolins. Figure 1A,B show that, Cav-1 S80V PC12 cells exposed to NGF developed a more extensive and denser neurite network than normal PC12 cells. This enhanced morphological differentiation was observed even at low concentrations of NGF. The NGF-induced morphological differentiation of Cav-1 S80V PC12 cells appears to be potentiated in comparison to normal PC12 cell response, as was observed with the differentiation of Cav-2 PC12 cells (Figure 1). Figure 2A shows that, following NGF exposure, Cav-1 S80V PC12 cells and normal PC12 cells stop proliferating with similar kinetics. Thus, expression of Cav-1 S80V does not result in the inhibition of the anti-mitogenic effect nor of the differentiation response that are observed with Cav-1 expression. Furthermore TrkA internalization is not affected by expression of Cav-1 S80V, in contrast to that of Cav-1 (Figure 3). Finally, in Cav-1 PC12 cells, the reduction of nuclear pRsk2, concomitant with the accumulation of pRsk2 at the cell periphery, is associated with an almost total inhibition of CREB phosphorylation. In contrast, NGF treatment of Cav-1 S80V PC12 cells results in nuclear localization of pRsk2 along with a high level of pCREB, superior to that observed in NGF treated normal PC12 cells (Figure 8A,B).

Taken together, these results show, that while expression of Cav-1 inhibits TrkA trafficking and NGF signaling, the Cav-1 S80V mutant does not, and actually potentiates the NGF response.

## 3. Discussion

The aim of this study was to gain insight into the mechanisms underlying the effects of membrane microdomain components Cav-1 and Cav-2 on growth factor signaling using the NGF receptors TrkA and p75^NTR^ as model.

A “ying and yang” dichotomy between Cav-1 and Cav-2 was observed, with Cav-1 inhibiting and Cav-2 potentiating the response to NGF. Cav-1 expression impaired exit from lipid rafts and internalization of NGF receptors, TrkA and p75^NTR^, without, however, abrogating TrkA short-term activation, nor downstream effector activation up to and including Rsk2. Remarkably, Cav-1 expression was associated with accumulation of activated Rsk2 at the plasma membrane and subsequent inhibition of CREB phosphorylation. These results indicate that Cav-1 is retaining the TrkA receptor signaling complex up to and including Rsk2 in the activated state at the plasma membrane, thereby preventing its nuclear translocation and phosphorylation of CREB.

By contrast, PC12 cells expressing Cav-2, displayed normal kinetics of cell-cycle arrest when exposed to NGF, while morphological differentiation was potentiated. In Cav-2 PC12 cells, NGF receptor exit from lipid rafts and internalization, as well as nuclear localization of pRsk2, were similar to that observed in PC12 cells. However, phosphorylated forms of TrkA effectors MAPK, Rsk2 and CREB, exhibited higher levels and/or longer activation kinetics. Taken together, these results demonstrate differential modulation of NGF signaling events by Cav-1 and Cav-2.

### 3.1. NGF-Induced Exit of TrkA and p75^NTR^ from Lipid Rafts

Numerous growth factor receptors have been observed to move in to or out of membrane microdomains [71,72], including neurotrophic factor receptors [73,74]. Cav-1 has been reported to play a role, independent of clathrin, in the internalization and sorting of several growth factor receptors including fibroblast growth factor receptor [75] and insulin receptor [76]. Here we show that NGF induces TrkA and p75^NTR^ exit from lipid rafts in PC12 cells. It has been reported that TrkA and TrkB reside outside lipid rafts prior to neurotrophic factor stimulation, then concentrate in lipid rafts consecutive to treatment with their respective ligands [77,78,79]. The case is similar for p75^NTR^ in sympathetic neurons [13]. By contrast, a number of studies have provided evidence that, in the absence of treatment, TrkA is enriched in lipid rafts [11,12,14,15,17,80], as observed herein.

The use of different methods for lipid raft isolation could explain the discrepancies. The studies presented herein offer insight for investigating the role of the caveolins in other experimental paradigms, of which growth factor signaling in particular, thereby contributing to our growing understanding of their diverse actions. Clearly, the very definition of growth factor binding-initiated translocation into, or out of lipid rafts, is dependent on the extraction protocol. While we, and others [11,12,14,17,80] used a detergent-independent method, either on post-mortem tissue or in cultured cells [13,77,78,79], used a Triton X100-dependent method, which has been shown to induce the formation of non-physiological structures [81]. This discrepancy notwithstanding, the common conclusion of these observations is that there is ligand-induced movement of the receptors between different membrane compartments. This underlines an interesting paradox that warrants clarification, which would offer insight on the microenvironment of the receptors in the different cellular membranes. The use of microscopy, and high-resolution microscopy, in particular [76,82,83], will help to substantiate the previous observations, and validate which extraction protocol(s) reflect what is actually occurring in and on the cell.

### 3.2. Cav-1 Mode of Action

Expression of Cav-1 in DRG, attenuated the differentiation response. The results presented herein confirm that, in PC12 cells, expression of Cav-1 inhibits NGF-induced morphological differentiation [14,16]. This is due therefore, to inhibition of NGF-induced TrkA and p75^NTR^ exit from lipid rafts by Cav-1. It has been proposed by Lajoie and Nabi 2007 that Cav-1 negatively regulates endocytosis either by stabilizing raft invagination at the cell surface or by sequestering key structural components, notably including cholesterol and dynamin [84,85], independently of the presence of caveolae [86]. The fact that pRsk2 accumulates at the periphery of the cell following a pattern closely resembling that of Cav-1, and TrkA (as illustrated in the dynamic experiments of Figure 5) suggests that Cav-1 is indeed sequestering the TrkA signaling complex at the cytoplasmic membrane in these cells. This effect is dependent on the presence of Serine 80, as demonstrated by the results obtained with the S80V mutant. The limited studies on this residue suggest a key role in regulating the binding of free cholesterol in cell membranes [87]. Whether this has an impact on Cav-1’s effect on TrkA trafficking remains to be determined.

Cav-1 has been reported to directly inhibit EGFR tyrosine kinase activity in vitro [88]. The results presented herein indicate that Cav-1 expression in PC12 cells results in the prevention of the anti-proliferative response, at least in part via the prevention of the induction of p21^Cip1/Waf1^. However, the NGF-TrkA, Ras, Raf, MAPK-dependent signaling pathway is not inhibited per se since TrkA, MAPK, and Rsk2 are indeed activated with similar kinetics in PC12 clonal lines stably-expressing Cav-1 as compared to PC12 cells in response to NGF. CREB phosphorylation, the triggering event essential for propagation of the anti-proliferative and differentiation responses, cannot occur since the activated Rsk2 is retained at the cell membrane.

### 3.3. MC192 Induced NGF Receptor Immobilization in Rafts

MC192 treatment mimics Cav-1 effects on internalization of TrkA [58]. MC192 binds to the extracellular domain of p75^NTR^, and without preventing the binding of NGF, attenuates NGF signaling via TrkA [57,89]. Application of MC192 to adult DRG resulted in a slight decrease in TrkA phosphorylation (30 min and 24 h) and yet was shown to significantly attenuate, if not block, neurite outgrowth [90]. The results presented herein offer novel insight into the mechanism via which MC192 is acting on NGF signaling. Namely, they indicate that MC192 prevents NGF-induced exit of both p75^NTR^ and TrkA from lipid rafts. This result suggests that the conformational change provoked (or prevented) by the binding of MC192 to p75^NTR^ directly impacts upon TrkA conformation, preventing p75^NTR^’s NGF-dependent exit from lipid rafts and, thus, internalization. The sequestration in lipid rafts therefore affords an initial explanation of the previously observed reduction of internalization of TrkA [58]. Further, this observation offers a novel means by which to study the dynamics of the intimate interactions between these two receptors that have been so elusive to the field.

### 3.4. Effect of Cav-2 on the NGF Signaling Pathway

Cav-2 expression in PC12 cells resulted in enhanced morphological differentiation. Very little is known about Cav-2, which has often been considered an accessory protein [91,92,93]. However Cav-2 has been shown to be directly involved in endocytosis of bacteria and apical trafficking of lipids [94,95]. Cav-2 is upregulated in esophageal and bladder carcinomas [96,97], which is consistent with the idea that Cav-2 could potentiate the MAPK pathway. Here, the potentiating effect of Cav-2 is associated with an increase and/or a longer duration of downstream effector activation as compared to normal PC12 cells.

Alternatively Cav-2 could promote TrkA signaling in a manner similar to that which has been observed for the insulin receptor (IR). Notably, Cav-2 phosphorylation prolonged IR activation by preventing its interaction with an inhibitory protein, SOCS-3 [98].

However the bulk of Cav-2 is not targeted to the plasma membrane and is localized in the Golgi apparatus [99]. This observation suggests that Cav-2 modulation of the NGF signaling pathway may not be mediated by the direct interaction between Cav-2 and TrkA at the plasma membrane. One possible mechanism by which Cav-2 could trigger its effects is via retaining a negative regulator of TrkA signaling in the Golgi apparatus, such as a phosphatase [100].

## 4. Materials and Methods

### 4.1. Reagents

Cell culture media is from Thermo Fisher Scientific (Courtaboeuf, France). Fetal bovine serum and horse serum were from Sigma (St. Quentin Fallavier, France) and GE-PAA (Velizy-Villacoublay, France), respectively. Transfection reagent lipofectamine 2000 is from Thermo Fisher. Dual luciferase kits are from Promega (Madison, WI, USA). ECL chemiluminescence system detection system is from GE-Amersham Pharmacia Biotech (Velizy-Villacoublay, France). ProLong Gold antifade reagent is from Invitrogen (Thermo Fischer Scientific, Courtabeuf, France) and Vectashield with DAPI is from Abcys (Courtaboeuf, France). NGF 2.5S Grade II from mouse submaxillary glands was purchased from Alomone Labs (Jerusalem, Israel). Anti-Histone H1 antibody is from Upstate Biotechnology (Lake Placid, NY, USA). Anti-TrkA extracellular domain (RTA) [56] was a kind gift of Louis F. Reichardt (University of California, San Francisco, CA, USA). Anti-p75^NTR^ carboxy-terminus serum is a kind gift of Moses. V. Chao (New York University Medical Center, New York, NY, USA). Anti-p75^NTR^ extracellular domain mAb MC192 is from Merck Millipore—Chemicon International (Fontenay sous Bois, France). Anti p21 mAb CP36 is a kind gift of Wade Harper (Baylor College of Medicine, Houston, TX, USA). Anti-Trk C-14, anti-phosphotyrosine (PY99), anti-pRsk2, and anti-Cav-1 antibodies are from Santa Cruz Biotechnology (Dallas, TX, USA). Anti-phospho-MAPK antibodies are from Promega. Anti-pCREB antibody is from Ozyme (Saint Quentin Yvelines, France). Anti-Cav-2 mAb is from Transduction Laboratories (Le Pont de Claix, France). Anti-β-tubulin mAb is from Sigma. Protein A Sepharose 4 Fast Flow is from Amersham Pharmacia. HRPO-linked anti-rabbit or anti-mouse IgG secondary antibody where purchased from GE Healthcare (Velizy-Villacoublay, France). Dylight Fluo-linked anti-rabbit or anti-mouse IgG secondary antibodies are from ThermoFisher Scientific. Alexa-Fluo linked anti-rabbit, anti-mouse and anti-goat antibodies are from Invitrogen. K252a is from Merck-Calbiochem (Fontenay sous Bois). The siRNA against caveolin-1 (sc-106996); caveolin-2 (sc-270431) and FITC-conjugated scrambled siRNA (sc-36869) are from Santa Cruz. siRNA transfection reagent lipofectamine RNAiMAX is from Life Technologies (Thermo Fisher Scientific, Courtaboeuf, France).

### 4.2. Cell Culture and Transfection

PC12 cells were grown as previously described [42]. cDNAs of human caveolin 1 (IMAGE #488533) and human caveolin 2 (IMAGE #491497) were purchased from Research Genetics (Huntsville, AL, USA). Caveolin 1 and 2 cDNAs were subcloned in the pcDNA3.1 (+) vector (Clontech) and correct cloning was verified by sequencing. PC12-Cav-1 and PC12-Cav-2 monoclonal populations were obtained by calcium-phosphate transfection of PC12 cells with pcDNA3.1-Cav-1 or pcDNA3.1-Cav-2 constructs, followed by an initial selection of 4 weeks in the presence of 0.4 mg/mL of G418 (Life Technologies). Antibiotic-resistant colonies were assayed for transgene expression by immunoblot analysis. Taking pcDNA3.1-Cav-1 plasmid as a template pcDNA3.1-Cav-1 S80V mutant was generated using QuickChange XL Site directed Mutagenesis Kit Agilent-Stratagene (Les Ulis, France) according to the manufacturer’s instruction. Primers were purchased from MWG-Biotech (Ebersberg, Germany) AG: S80V mutant: sense: 5′-CCA GAA GGG ACA CAC **GTG** TTT GAC GGC ATT TGG AAG GCC AGC-3′, anti-sense: 5′-GCT GGC CTT CCA AAT GCC GTC AAA **CAC** GTG TGT CCC TTC TGG-3′ (codon in bold is the position 80). PC12-Cav-1 S80V monoclonal populations were obtained by lipofectamine 2000 transfection of PC12 cells with pcDNA3.1-Cav-1 S80V constructs, followed by an initial selection of four weeks in the presence of 0.8 mg/mL of G418 (Life Technologies). Antibiotic-resistant colonies were assayed for transgene expression by immunoblot analysis.

### 4.3. siRNA Transfection

2 × 10^4^ cells were seeded per well onto 24-well plates in culture medium 8–12 h before transfection. The transfection of siRNA at a final concentration of 30 nM was performed using Lipofectamine RNAiMAX according to the manufacturer’s instructions. FITC-conjugated scrambled siRNA was used as a non-targeting control siRNA. Medium (without serum) was refreshed 8–12 h after transfection, cells were then maintained in DMEM medium supplemented with 0 or 20 ng/mL NGF for 72 h before imaging. The neurite outgrowth was quantified using NeuronJ plug-in for ImageJ (National Institutes of Health, Bethesda, MD, USA). Cells were considered “with neuritis” when neurites were ≥3 cell diameters. Statistical significance was tested with the two-tail unpaired Student’s *t*-test.

### 4.4. DRG Neuron Culture, Transfection, and Quantification

All procedures were performed in accordance with French and European legislation on animal experimentation. Primary DRG neurons were prepared from E14.5 mouse embryos and electroporated as previously described [37] with pEGFP (Clontech, Saint-Germain-en-Laye, France) (0.5 µg) and pCDNA3.1-Cav-1 fused to RFP (gift from Ari Helenius, Addgene Plasmid 14434) [38] or pCDNA3.1-RFP (1 µg) endotoxin free plasmids (XtraMaxi, Nucleobond, Macherey Nagel, Hoerdt, France). Control RFP was derived from the Cav-1-RFP plasmid. The Cav-1 coding sequence was removed by BamHI and Not1 digestion and plasmid ligation was performed after Kleenow fill-in. Because of the low number of cells required, both control and Cav-1 electroporations could be performed on the same batch of dissociated cells.

Electroporated cells were plated on poly-l-Polylysine and laminin coated coverslips (PLL: 50 µg/mL; laminin: 10 µg/µL, Sigma. 1 h after plating, medium (F12 + 10% FCS) was complemented with 50 ng/mL NGF (N0513, Sigma) and antibiotics (1% final, 15140, Gibco, Courtaboeuf, France). 10 µM of AraC was added the next morning (C1768, Sigma).

Control and caveolin-1 cultures from the same dissociation batch were fixed at different time points in 2% paraformaldehyde at 4 °C and neurite growth was analyzed. Four montages of nine images were collected in different regions of the coverslips to minimize bias due to different cell density. Number of transfected neurons per image was calculated and neurite length were quantified from the GFP channel in ImageJ (National Institutes of Health, Bethesda, MD, USA; http://imagej.nih.gov/ij) using NeuronJ plugin [101]. Statistical significance was tested with the two-tail unpaired Student’s *t*-test.

### 4.5. Western Blot Analysis

To obtain lysates, cells were washed once and collected in ice cold phosphate-buffered saline (PBS). Pelleted cells were resuspended in lysis buffer (20 mM tris-HCl, pH 8.0; 137 mM NaCl; 2 mM EDTA; 10% glycerol; 1% Nonidet P-40; 20 µM leupeptin; 1 mM sodium vanadate; 1 mM Pefabloc; 0.15 U/mL aprotinin; 1 mM ß-glycerophosphate; 3 mM sodium-fluoride). After 30 min incubation on a rotating wheel at 4 °C, the extracts were clarified by centrifugation at 12,000× *g* for 20 min. The protein concentration of the supernatants was quantitated using the DC (detergent compatible) protein assay (Bio-Rad, Marnes-la-Coquette, France). Next, 5X-SDS-PAGE sample buffer was added to the lysates prior to boiling for 5 min. Proteins were separated by SDS-PAGE and transferred to nitrocellulose membranes. Membranes were blocked in TBST (25 mM Tris-HCl pH 7.4, 137 mM NaCl, 3 mM KCl, 0.1% Tween-20) containing 5% (*w*/*v*) dried milk or 5% (*w*/*v*) bovine serum albumin (BSA) (sigma) depending on the primary antibody, incubated 1 h at room temperature with primary antibody diluted in TBST with 5% BSA. After two washes with TBST, membranes were incubated 1 h with horseradish peroxidase-conjugated secondary antibodies, washed twice with TBST and immunoreactive proteins were visualized using chemiluminescence.

Quantification of the scanned gels was performed using the Gel analysis routine of ImageJ either with specification of individual lanes or selection of the entire row as described in the online documentation. The values obtained were normalized for those from the corresponding tubulin loading control and, subsequently, to the maximum signal in a given series, in order to be able to compare results obtained from multiple experiments. Statistical significance between selected conditions was ascertained with the unpaired, two-tail Student’s *t*-test.

Alternatively, to obtain quantitative results in some experiments, protein expression was evaluated by SDS-PAGE followed with a western blot analysis using the Odyssey. In this case, membranes were blocked in TBS (25 mM Tris-HCl pH 7.4, 137 mM NaCl, 3 mM KCl) containing 5% (*w*/*v*) BSA, incubated 1 h at room temperature with primary antibody diluted in TBST with 5% BSA. After four washes with TBST, membranes were incubated 1 h with Dylight Fluo-linked secondary antibody allowing quantitative infrared fluorescence detection using Odyssey imaging system Odyssey (ODY-1092, ScienceTec, Villebon-sur-Yvette, France).

### 4.6. Luciferase Assays

Wild type PC12 cells, Cav-1 PC12 cells and Cav-2 PC12 cells were spread on collagen/poly L-lysine-coated 6 well dishes [58] and transfected with lipofectamine 2000 with 2 µg of p21P93SLuc reporter as previously described [45,102] and 0.1 µg of the reporter pEGFP-C2 or alternatively with 0.1 µg of pCMV H-RasV12. 24 h after transfection, cells were transferred to 96-well plates, and treated or not with 50 ng/mL of NGF. 48 h later, cells were lysed and assayed for luciferase activity using the Dual Luciferase kit (Promega).

### 4.7. Immunoprecipitation

Following NGF treatment, cells were collected and lysed. Cleared lysates (1 mg protein per immunoprecipitate) were then incubated on a rotating wheel for 2 h at 4 °C with 2 µg of Trk C-14 antibody. Protein A Sepharose beads were added to bind the antibodies; after 2 h at 4 °C beads were washed for times with lysis buffer and proteins were eluted by boiling for 5 min in sample buffer.

### 4.8. Isolation of Lipid Rafts

Lipid rafts (LR) were isolated essentially as described [103]. Briefly, PC12 cells from a 162 cm^2^ cell culture flask grown to 60–70% confluence were collected in ice-cold PBS and resuspended in 1.5 mL of Na_2_CO_3_ 0.5 M pH 11.0. Homogenization was carried out using a sonicator (30 three-second bursts on ice; Vibra Cells, Sonics & Materials, Newtown, CT, USA). The homogenate (6 mg protein/1.5 mL) was then adjusted to 45% sucrose by addition of 1.5 mL of 90% sucrose prepared in H_2_O and loaded in a Beckman ultracentrifuge tube under a 5–35% discontinuous sucrose gradient (3 mL of 5% sucrose/6 mL of 35% sucrose; both in 250 mM Na_2_CO_3_ pH 11.0). After centrifugation at 39,000 rpm for 14 h in a SW41 rotor (Beckman, Villepinte, France), lipid rafts were apparent as a light-scattering band confined to the 5–35% sucrose interface. Twelve fraction of 1 mL were collected by pipetting from the top of the gradient 20 µL were used for SDS-PAGE. The lipid rafts were present in fractions 3 and 4.

### 4.9. Confocal Immunofluorescence Microscopy

PC12 cells were spread on collagen/poly l-lysine-coated cover slips [58]. Cells were rinsed twice with PBS, fixed 30 min in PBS 3.7% formaldehyde and permeabilized one minute in PBS 0.5% Triton X-100. After washing with PBS, cells were blocked with PBS 0.5% BSA for 30 min and incubated 1 h with primary antibodies diluted in blocking buffer. Cells were washed three times in PBS and incubated 1 h with secondary antibodies conjugated to Alexa 488, Alexa 555, or Alexa 647. Cells were washed again three times in PBS and coverslips were mounted with ProLong Gold antifade or Vectashield with DAPI reagent. Scanning fluorescence images were acquired using the DM6000-confocal unit coupled to a Leica (Nanterre, France) spectral confocal TCS SP5 AOBS with a HCX Plan APO 63/1.2 W Corr oil-immersion objective. Data acquisition was performed with Leica LAS AF SP5 software. Quantitation of the fluorescence was assessed using ImageJ software. For the study of TrkA internalization, cells were transiently transfected with a TrkA-EGFP fusion protein expression vector [55] using lipofectamine 2000, and immunostaining was performed 48 h post-transfection. Cells were preincubated for 15 min at 4 °C with RTA antibody (1/500 dilution). Cells were then replaced at 37 °C for 20 min prior to standard fixation and immunostaining procedure as described above. Scanning fluorescence images were acquired using the MRC1000-confocal laser unit (Bio-Rad Labs, Hercules, CA, USA) coupled to a Zeiss (Marly-le-Roi, France) Axioplan Microscope equipped with a Zeiss Plan-Apochromat 63X/1.4 oil-immersion objective, an LSM 510 camera. Data acquisition was performed with LSM510 software.

### 4.10. Statistical Analysis

Averages and standard errors of the mean were calculated and analyzed for statistical significance vs. controls, using the unpaired, two-tail Student’s *t*-test (Figure 1H, Figure 2B,C Figure 4B, Figure 6B, Figure 7B and Figure 9B). For Figure 3C, the regular two-way ANOVA was applied twice using Prism^©^ 5 for Windows^®^, version 5.01. First ± NGF was compared in each cell type followed by the Bonferroni correction *post hoc* test (* *p* values). The second pass compared—NGF between cell types and + NGF between cell types, again followed by the Bonferroni correction *post hoc* test (# *p* values). For Figure 8D–F, the two-way ANOVA test with the Bonferroni *post hoc* test was applied via Microsoft^®^ Excel^©^. For Mac 2011, version 14.5.5 (150821) with StatPlusMac^®^ (AnalystSoft, Walnut, CA, USA).

## 5. Conclusions

The data presented herein provide evidence for a differential role of Cav-1 and Cav-2 on NGF signaling. Caveolins are key components of NGF receptor microenvironment that play an important role in the outcome of NGF signaling. Cav-1 inhibits NGF-induced cell responses acting on the trafficking of p75 ^NTR^, TrkA, and its downstream effectors by sequestration of key signaling molecules at the cell membrane, resulting in the prevention of their nuclear translocation and phosphorylation of a key transcription factor CREB. Cav-1 S80V, a non-phosphorylatable form of Cav-1, does not present the same inhibitory effects as Cav-1 on TrkA trafficking and downstream signaling, suggesting that phosphorylation of this residue is key to this effect. It contributes to understanding the molecular impact of specific Cav-1 mutations in certain cancers where TrkA, p75^NTR^, and NGF are involved in the tumorigenic response, while raising the question of the potential impact on trafficking of other receptors and channels. Cav-2 has the opposite effect to Cav-1, enhancing the cellular response to NGF. This highlights the opportunity for gaining a better understanding of the impact of major lipid raft components on NGF receptor trafficking and subsequent NGF signaling in the context of cancer and neuronal differentiation.

## Supplementary Materials

Supplementary materials can be found at www.mdpi.com/1422-0067/18/4/693/s1.
