# Supplementary material for: Nerve Growth Factor Signaling from Membrane Microdomains to the Nucleus: Differential Regulation by Caveolins
Source: Int J Mol Sci. 2017 Mar 24;18(4):693. doi: 10.3390/ijms18040693 (PMC5412279; doi:10.3390/ijms18040693)
Supplement: Supplementary file 1 [file ijms-18-00693-s001.pdf]

Supplementary Materials: Nerve Growth Factor Signaling from Membrane Microdomains to the Nucleus: Differential Regulation by Caveolins

Ambre Spencer, Lingli Yu, Vincent Guili, Florie Reynaud, YinDi Ding, Ji Ma, Jérôme Jullien, David Koubi, Emmanuel Gauthier, David Cluet, Julien Falk, Valérie Castellani, Chonggang Yuan and Brian B. Rudkin

Table S1. Summary of the experiments performed on different clones of PC12 cells stably expressing Cav-1 or Cav-2. Analyses of the different clones, performed as described in the Materials and Methods, yielded comparable results.

	CAV-1							CAV-2				
**Clone**	3	11	12	16	18	20	**Clone**	5	11	13	14	23
**Proliferation**			+	+			**Proliferation**	+				+
**Differentiation**	+	+	+	+	+	+	**Differentiation**	+	+	+	+	+
**Western**							**Western**					
Cav-1	++	+++	+	++	+++	+++	Cav-2	++	++	+++	++	+
TrkA PO4			+	+	+	+	TrkA PO4	+		+	+	+
pMAPK 42/44	+	+	+	+	+		pMAPK 42/44	+	+			+
pRSK	+	+		+	+	+	pRSK	+	+			
pCREB/CREB	+	+		+	+	+	pCREB/CREB	+	+			
p38				+			p38	+				
pAkt	+			+			pAkt	+	+			
pJNK	+			+			pJNK	+	+			
**LRF**		+		+		+	**LRF**	+	+			+
**IF**		+		+		+	**IF**	+	+			

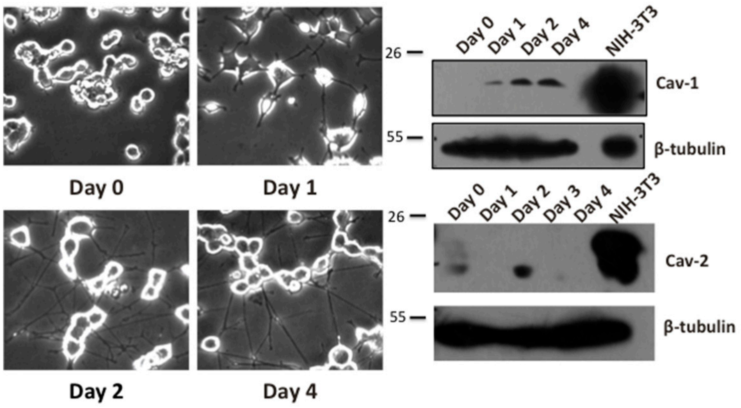

Figure S1. Expression of endogenous Cav-1 and Cav-2 in response to NGF. PC12 cells were exposed to NGF (50 ng/mL), harvested, and extracted for Western blot analysis for Cav-1 and Cav-2 as described in the Materials and Methods. Exponentially growing NIH-3T3 cells are used as a positive control. Beta-tubulin is used as loading control.

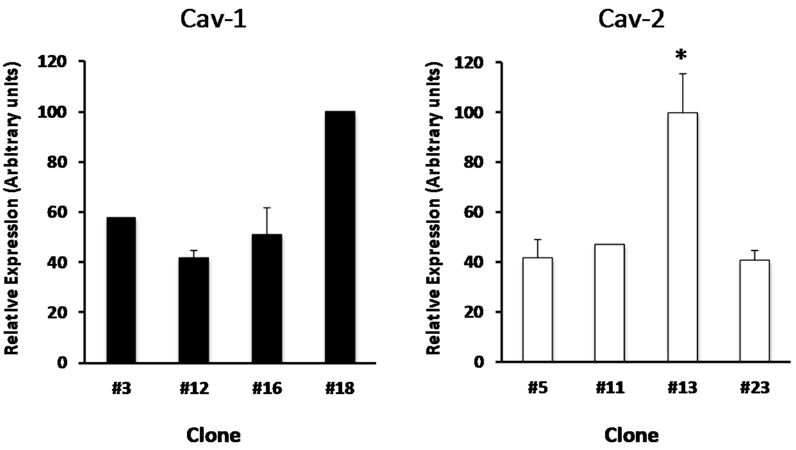

Figure S2. Relative expression levels of Cav-1 and Cav-2 in selected clones. PC12 clones expressing Cav-1 or Cav-2 were extracted for Western blot analysis and quantified using the gel analysis function of ImageJ as described in the Materials and Methods. Beta-tubulin was used as the loading control. The clone expressing the highest relative amount was considered 100%. (Mean ± SEM derived from eight gels generated in four independent experiments. Statistical significance was ascertained using the unpaired, two-tail Student's *t*-Test comparing with clone Cav-1 #12 and Cav-2 #11 respectively. * $p < 0.05$.)
